# Special Issue: Molecular Mechanisms of Bioactive Nutrients Promoting Human Health

**DOI:** 10.3390/ijms26189184

**Published:** 2025-09-20

**Authors:** Baojun Xu

**Affiliations:** Food Science and Technology Program, Department of Life Sciences, Beijing Normal-Hong Kong Baptist University, Zhuhai 519087, China; baojunxu@uic.edu.cn

Communicable and non-communicable diseases are major contributors to the global burden of disease. Although their etiologies differ, these diseases share molecular mechanisms, including cellular oxidative stress damage, inflammation, and dysregulated immune signaling. In this context, a growing body of evidence suggests that bioactive nutrients of dietary and microbial origin—such as vitamins, phytochemicals, fatty acids, prebiotics, and probiotics—can mitigate the impact of infectious illnesses and lower the risk of non-communicable diseases by modulating oxidative and inflammatory pathways, shaping the gut microbiota, influencing metabolic and endocrine signaling, and activating immune responses to support human health [1,2]. However, the underlying molecular mechanisms by which these bioactive compounds regulate cellular signaling and adaptively reshape tissue homeostasis in humans remain to be elucidated.

This Special Issue of the *International Journal of Molecular Sciences*, titled “Molecular Mechanisms of Bioactive Nutrients Promoting Human Health,” includes eleven contributions spanning in vitro systems, animal models, human studies, and review papers. They offer mechanistic insights into how bioactive nutrients promote human health and propose promising strategies to prevent or treat diseases. For instance, Cocksedge et al. reviewed recent preclinical and clinical findings on superoxide dismutase (SOD)-rich *Tetraselmis chuii*, indicating that it may act as an indirect antioxidant, modulate inflammatory pathways, and prevent immune and mitochondrial dysfunction by activating NRF2 and SIRT1 [Contribution 1]. Yan et al. collected evidence on the dietary flavonoids vitexin and isovitexin, illustrating their cardiovascular, glycemic, anti-obesity, anticancer, antioxidant, anti-inflammatory, and neuroprotective properties. They also emphasize the urgent need to define molecular targets and signaling networks, and to establish clinically effective and physiologically relevant doses [Contribution 2]. Han et al. used the 3T3-L1 adipocytes model to investigate the anti-obesity effect of *Lactobacillus brevis*–fermented *γ*-aminobutyric acid, showing that it suppresses adipogenesis and lipogenesis, induces lipolysis via fatty acid oxidation, and enhances energy expenditure via UCP1-mediated browning, all of which support its potential as a novel anti-obesity functional food [Contribution 3].

Colon cancer (CRC) is the second leading cause of cancer-related deaths worldwide, driving interest in bioactive nutrient-based interventions with therapeutic potential and fewer side effects. In a comprehensive review, Bentharavithana et al. summarized the anticancer effects, particularly the anti-colon cancer properties, of edible medicinal mushrooms. The compounds derived from these medicinal mushrooms, including terpenoids, phenols, polysaccharides, ascorbic acid, glycosides, and organic acids, can inhibit proliferation, induce apoptosis, and reduce inflammation, thereby contributing to the prevention and treatment of colon cancer. The authors emphasize the need for rigorous human clinical trials to establish efficacy and safety before clinical adoption [Contribution 4]. Moreover, studies using an azoxymethane (AOM)-induced CRC rat model showed that low-molar-mass oat *β*-glucan supplementation improved colonic redox balance, reduced inflammation, and decreased lipid peroxidation. Wilczak et al. further demonstrated that this *β*-glucan facilitates colonic metabolism remodeling during early carcinogenesis, as evidenced by alterations in the amino acid, purine, biotin, and folate pathways [Contribution 5]. Similarly, Yang et al. reported that chicoric acid alleviated dextran sulfate sodium (DSS)-induced colitis in mice by reducing pro-inflammatory cytokines (TNF-α and IL-6), modifying the gut microbiota (by decreasing *Bacteroidetes* and increasing *Lachnospiraceae*), and improving metabolic homeostasis (by restoring thiamine and lithocholic acid) [Contribution 6]. In an in vivo study using two murine colorectal cancer models (the orthotopic MC-38 cecum injection model and the inflammation-driven AOM/DSS model), probiotic efficacy was found to be model-dependent. *Lactobacillus*/*Bifidobacterium* mix (CI) reduced tumor burden in the orthotopic MC-38 model, while *Bifidobacterium* alone (CII) suppressed inflammation and tumors in the AOM/DSS model. Collectively, these findings support context-specific, microbiota- and diet-based strategies for CRC prevention and treatment and encourage the development of tailored probiotic strategies [Contribution 7].

Neagu et al. reviewed the interplay between diet, dietary patterns, behaviors, chemical xenobiotic exposures, and breast cancer, highlighting evidence that a healthy diet pattern with appropriate nutritional behaviors activates anti-tumor pathways and suppresses tumor progression, while the accumulation of foodborne contaminants can synergistically promote tumorigenesis [Contribution 8]. Jędrzejewski et al. investigated a combination strategy by pairing *Coriolus versicolor* (CV) with the inhibitor of the phosphatidylinositol 3-kinase (PI3K) signaling pathway, LY294002. In MCF-7, HeLa, and A549 cell models, co-treatment decreased viability and colony formation, induced G0/G1 arrest and apoptosis, and inhibited migration/invasion. While this additive cytotoxic approach shows promise, the authors stress the need to evaluate pharmacokinetics, bioavailability, safety, and the nature of interaction in animal studies [Contribution 9].

In addition to the research on bioactive nutrients in non-communicable diseases, this Special Issue also features two timely studies on antiviral immunity. One study assessed whether an enzymatically liberated salmon oil can increase immune recovery after acute SARS-CoV-2 infection by regulating cytokine, chemokine, and interferon-related gene expression; the other study combined bioinformatics analysis with in vitro testing to evaluate the anti-influenza A effect of *Tagetes erecta* Linn. (TE) extract. In a randomized pilot study of adults with mild to moderate COVID-19 (*n* = 11), Currie et al. found that participants receiving best supportive care and 4 g/day of full-spectrum, enzymatically liberated salmon oil for 28 days experienced reduced inflammation, improved interferon response, increased lung barrier function, and stronger immune memory [Contribution 10]. However, larger and well-controlled clinical trials are required to determine whether these findings translate into meaningful clinical outcomes. In addition, Kim et al. integrated bioinformatics, molecular docking, antiviral assays, and plaque reduction tests, identifying lutein as a key active component in the TE extract and IL-6 as a central hub target associated with influenza. In conclusion, these contributions demonstrate how nutrition-derived interventions impact immune pathways to enhance human health, though more rigorous clinical validation of efficacy, safety, and dosing is required [Contribution 11].

This Special Issue of the *International Journal of Molecular Sciences* integrates the latest in vitro, animal, and human studies across non-communicable diseases and antiviral immunity, revealing the mechanisms through which bioactive nutrients promote human health. The research contributions to this Special Issue demonstrate the growing potential of dietary interventions in disease prevention and treatment. Although further steps are still necessary for clinical translation, such as optimizing dose and delivery and conducting comprehensive safety assessments, ongoing advances in this rapidly evolving field are promising.

## Data Availability

Not applicable.
